# Notes and News

**Published:** 1902-05-24

**Authors:** 


### Notes and News.

No student under sixteen years of age will be admitted to the Medical School of the Aberdeen University.

The Duke of Northumberland, Vice-President, will preside at the Sanitary Institute Coronation Dinner to be held on Monday, June 2nd, at the Midland Grand Hotel.

It is announced that Professor Virchow has resigned the presidency of the Berlin Medical Society. Leave of absence has been granted him from his University duties during the summer term.

A generous donation of £1,000 has been offered to the Royal Eye and Ear Hospital, in Dublin, provided that £4,000 is forthcoming in November next from other sources. The hospital is being rebuilt.

The Birmingham and Midland Homœopathic Hospital has been altered and extended and is now open to the reception of patients once more. The cost of the improvements has been £5,000. There is now accommodation for 50 patients, and the hospital contains an operating theatre and three private wards.

A temporary small-pox hospital is being erected at Hanworth, Middlesex. It appears that the surface water from the site of this hospital drains into the Thames immediately above the intakes of the Southwark and Vauxhall Water Company, the Grand Junction Company, and the West Middlesex Water Company, and the ground is sometimes under water from four to nine inches.

In a letter to the *Times* on the subject of pay wards in hospitals Dr. Robert Saundby, of Birmingham, says:—"If Sir Henry Burdett is able to devise a plan by which the pay wards of a hospital can be made truly self-supporting by payments within the compass of the purses of the artisan, clerk, shopman or shopwoman, without robbing either the charity or the medical staff, he will have earned an additional title to the gratitude of the public as well as of the medical profession."

We regret that Mrs. Sam Lewis was inadvertently referred to as Mrs. Lewis Waller in our columns in connection with her generous contribution to King Edward's Hospital Fund.

At the Westminster Hospital post-graduate clinical course Dr. Colcott Fox will give a demonstration on "Tuberculides of the Skin," with cases, on Tuesday, 27th inst., at 4.30 p.m.

Sir Frederick Treves has chosen for his subject for the Cavendish Lecture before the West London Medico-Chirurgical Society "Certain Phases of Appendicitis." The lecture will be given on Friday, June 20th.

His Grace the Duke of Fife, K.T., will take the chair at the fiftieth annual general court of Governors of the Hospital for Sick Children, Great Ormond Street, to be held in the Board Room, on Wednesday, May 28th, at 5 p.m. precisely.

The horrible and absolutely useless death of a young "parachutist"—as clear a case of the wanton sacrifice of a life for the mere amusement of an excitement-loving crowd as ever occurred in gladiatorial days—makes us ask again what is the use of civilisation and education if such brutal exhibitions are their product?

The considerations, now of long standing, to reorganise the hospital system of Paris have resulted in a proposal which embraces the rebuilding of a number of hospitals, and the enlargement and improvement of many others, at an estimated cost of 83,000,000 francs. By this means upwards of one thousand extra beds will be provided for the sick poor of Paris.

The authorities of the Westminster Hospital have applied for a summons against the Middlesex County Council, the ground of complaint being that the Council has commenced to erect a stand by the Middlesex Guildhall, directly in front of a portion of the hospital, for the purpose of providing seats from which to view the Coronation procession. The magistrate granted the process.

At the annual meeting of the British Medical Temperance Association, held on May 20, Professor Sims Woodhead, the president, being in the chair, the report showed that the members numbered 520, the student associates 436, and the associates 3. During the year the Council had arranged for a series of meetings intended to influence medical students. In view of the extension of the practice of total abstinence among medical professors and practitioners, both in this country, on the Continent, and in America, the council decided to communicate with the council of the American Medical Temperance Association, with that of the Society of Abstaining German Physicians, and with Dr. Lefrain on behalf of France, and proposed an international medical manifesto against the use of alcohol, which was to be signed by none except those who were themselves practising total abstinence. The signatures of about 200 British and Irish practitioners had already been received.

